# Exploring the Impact of Salicylic Acid and Farmyard Manure on Soil Rhizospheric Properties and Cadmium Stress Alleviation in Maize (*Zea mays* L.)

**DOI:** 10.3390/plants12173115

**Published:** 2023-08-30

**Authors:** Hafiz Haider Ali, Nimra Shehzadi, Muhammad Saqlain Zaheer, Mahmoud F. Seleiman, Khalid J. Aldhuwaib, Waqas ud Din Khan, Ali Raza

**Affiliations:** 1 Department of Agriculture, Government College University, Lahore 54000, Pakistan; dr.waqasuddin@gcu.edu.pk; 2 Sustainable Development Study Center (SDSC), Government College University, Lahore 54000, Pakistan; nimrazafar45@gmail.com; 3 Department of Agricultural Engineering, Khwaja Fareed University of Engineering and Information Technology, Rahim Yar Khan 64200, Pakistan; 4 Department of Plant Production, College of Food and Agriculture Sciences, King Saud University, P.O. Box 2460, Riyadh 11451, Saudi Arabia; mseleiman@ksu.edu.sa; 5 School of Biological Sciences, University of Reading, Reading RG6 6EX, UK; k.j.h.al-dhuwaib@pgr.reading.ac.uk; 6 Department of Agronomy, University of Sargodha, Sargodha 40100, Pakistan; aliraza7342@gmail.com

**Keywords:** salicylic acid, farmyard manure, rhizospheric properties, cadmium stress, maize, antioxidant enzymes

## Abstract

Cadmium (Cd) pollution is a growing environmental problem that negatively impacts plant growth and development, particularly in maize. In this research, the impact of farmyard manure (FYM) and salicylic acid (SA) on rhizospheric characteristics and the reduction of Cd stress in maize was examined at Government College (GC) University, Lahore, in 2022. The experiment was arranged with a randomized design, including three replications of 12 treatments (T_1_ = Control; T_2_ = Farmyard manure; T_3_ = Salicylic Acid; T_4_ = 100 mg/kg of soil Cd; T_5_ = 200 mg/kg of soil Cd; T_6_ = Farmyard manure + Salicylic acid; T_7_ = FYM + 100 mg/kg soil Cd; T_8_ = FYM + 200 mg/kg soil Cd; T_9_ = SA + 100 mg/kg soil Cd; T_10_ = SA + 200 mg/kg soil Cd; T_11_ = FYM + SA + 100 mg/kg soil Cd; T_12_ = FYM + SA + 200 mg/kg soil Cd). Results demonstrated that Cd stress negatively affected the maize plant and soil properties, but the application of SA and FYM was effective to mitigate the Cd stress up to a certain level. A reduction of 41.52%, 39.14%, and 39.94% in root length, length of the leaf, and crop growth rate was noticed, due to the Cd stress at 200 mg/kg soil, but this reduction was reduced to 18.83%, 10.35%, and 12.26%, respectively, when FYM and salicylic acid were applied as a combined application under the same stress level of Cd. The root biomass, leaf surface area, and length were all improved by SA and FYM, which enhanced the plant’s capacity to absorb nutrients and improve growth under Cd stress. In conclusion, the use of salicylic acid together with farm manure can be an effective approach to mitigate Cd stress in maize crops.

## 1. Introduction

Industrial and agricultural practices contribute to the accumulation of cadmium (Cd) in the soil. Plant growth and development are negatively impacted by Cd pollution, a serious environmental issue. Some of the physiological and biochemical alterations that plants undergo in response to Cd stress include a reduction in photosynthesis, increased oxidative stress, and a rebalancing of nutrients [1,2]. *Zea mays* L. is a staple grain crop, but its growth and productivity are severely affected by Cd stress. Therefore, it is essential to develop strategies to alleviate abiotic stress such heavy metals in maize and improve sustainable production in the agriculture sector [3,4]. In Pakistan, less maize is consumed directly by people, but its use in the feed and wet milling sectors is expanding far more quickly than anyone anticipated [5,6]. Cd-contaminated soils cannot provide the nutrients that maize requires from soil due to its high-nutrient requirements, which limits plant growth and prevents maize from reaching its maximum growth potential [7,8]. The absence of efficient production technologies and pest management methods, as well as moisture stress, low fertility, and poor cultural practices, are a few causes of the low yield [3].

Heavy metal pollution is going to increase on a dangerous level due to the escalating environmental crisis. Metropolitan areas of Pakistan are found to be contaminated with heavy metal pollution [9]. Industrial effluents contain chemicals and heavy metals that are released into water bodies without pretreatment. The groundwater, vegetation, and air have been found to be contaminated with heavy metals [10,11]. Acid rain, organic debris, and heavy metals are the main sources of soil contamination [12]. Heavy metals are particularly worrisome due to their non-degradability, high-toxicity, cumulative effects, and carcinogenicity. Heavy metals naturally occur in soil in trace levels, but their regional distribution poses problems owing to both natural and anthropogenic reasons [13,14].

Salicylic acid (SA) and farmyard manure (FYM) are potential candidates for alleviating Cd stress in plants. Salicylic acid (SA) plays a very important role in reducing metal stress such as Cr, Cd, and Cu [15]. Salicylic acid, at lower rates in plants, has the same effect as acclimatization and enhances the formation of antioxidants such as H_2_O_2_ [16]. Greater antioxidant enzyme production in plants can also improve plant growth under stress conditions [17]. Salicylic acid has the ability to mitigate a-biotic stress in crop plants by inducing positive physiological changes such as enhancing the water content in leaves, and improving nutrient uptake and the production of growth hormones [18]. SA is essential for the growth and development of plants. It can also reduce the harmful effects of Cd stress in a variety of plant species, including maize [8,14,19,20].

FYM is an organic supplement that can improve the soil’s physical, chemical, and biological qualities. FYM also provides nutrients and helps to improve the microbial activities in the soil that directly enhance the soil fertility level [21]. FYM application makes Cd less dangerous and helps plants to grow better under Cd stress [22]. FYM also enhances the organic matter contents, soil aeration, and nutrient availability in the soil [23]. Use of farmyard manure in crop production improves the soil structure, which creates a better environment for root growth. Farmyard manure also increases the soil’s ability to store water [21,24]. Farmyard manure aids in the growth of soil bacteria and their activities, which are critical for the easy availability of complex nutrients to plants [23].

Rhizospheric properties are crucial for plant growth and development. The rhizosphere is the soil region influenced by plant roots, which contains a complex microbial community that interacts with the plant [25]. Rhizospheric properties, such as soil pH, organic matter content, and microbial diversity, affect the availability of nutrients and the mobility of heavy metals in the soil. Therefore, improving the rhizospheric properties can enhance the plant’s tolerance to Cd stress [26]. Heavy metals in agricultural soils are a major environmental concern because of the longstanding toxicity and bioaccumulation of metals. Heavy metal toxicity can damage the beneficial microbes in the soil, creating water and soil pollution [27].

The high levels of Cd in maize crops slows down growth and photosynthetic activity, which lowers crop output [7]. To overcome these losses, a study is designed in which plant growth hormone “salicylic acid” and “farmyard manure” applications are used in the soil in maize crops. This study was planned with the objectives to examine the impact of SA and FYM on rhizospheric characteristics and to mitigate Cd stress in maize. 

## 2. Materials and Methods

### 2.1. Crop Growth Conditions

A planned pot experiment was arranged at the Government College (GC) University Lahore to investigate the effect of farmyard manure and salicylic acid on cadmium-induced physiological damage in maize crops. The experiment was conducted with a complete randomized design (CRD), having three replications. The Punjab Seed Corporation-approved maize variety (MMRI Yellow) was used in the experiment. These seeds were planted in 2 kgs of pot soil and farmyard manure, in accordance with procedures. Salicylic acid was obtained from the university laboratory and FYM was obtained from the nearest livestock farm. Table 1 lists the characteristics of the loamy soil that was utilized for the experiment.

Maize seeds were sterilized with 70% ethanol for 2 min, followed by 5% sodium hypochlorite for 30 min, and then rinsed with distilled water before being sown and transplanted into pots. The planting was done on March 22, 2022. The pots received good irrigation and the required fertilizer dosage (120-80-60 NPK kg ha^−1^) was used. 

The treatments applied in this experiment were as follows: T_1_ = control; T_2_ = Farmyard manure; T_3_ = Salicylic acid; T_4_ = 100 mg/kg of soil Cd; T_5_ = 200 mg/kg of soil Cd; T_6_ = Farmyard manure + Salicylic acid; T_7_ = FYM + 100 mg/kg soil Cd; T_8_ = FYM + 200 mg/kg soil Cd; T_9_ = SA + 100 mg/kg soil Cd; T_10_ = SA + 200 mg/kg soil Cd; T_11_ = FYM + SA + 100 mg/kg soil Cd; T_12_ = FYM + SA + 200 mg/kg soil Cd. 

### 2.2. Measured Parameters

Crop physiological and growth-related parameters, such as plant height (cm), plant weight (g), leaf length (cm), number of leaves (n), chlorophyll content, and NPK uptake (%) of the maize crop was determined after 30 days of sowing. Chlorophyll contents were noticed by using the procedure described by Schagerl and Künzl [28]. The absorbance of the sample was measured at 663 and 645 nm, using a UV/visible spectrophotometer (Spectro scan 80D, Kyoto, Japan). Chlorophyll concentration a&b was determined by using the following equation and procedure, as described by Porra [29].

Ch. a (mg mL^−1^) = (11.64 × A663) − (2.16 × 645)

Ch. b (mg mL^−1^) = (−3.94 × A663) + (20.97 × A645)

To determine the relative water content (RWC) of a leaf, a 0.5 g leaf sample was kept in the dark in distilled water at 4 °C for 24 h. Subsequently, the samples were dried in an oven at 65 °C for 48 h to obtain the dry weight (in grams), which was measured and recorded. The calculation of RWC was carried out using the formula described by Barr and Weatherley [30]:

RWC (%) = [(FW − DW)/(TW − DW)] × 100.

Crop growth rate (CGR) was calculated as described by Hunt [31]:

CGR = (W_2_ − W_1_)/(t_2_ − t_1_)

Net assimilation rate (NAR) was calculated using a method reported by Hunt [26]:

TDM/LAD = NAR

where, LAD stands for leaf area duration, and TDM stands for total dry matter.

The method described by Smolander and Kitunen [32] was used to measure the dissolved organic nitrogen (DON) and carbon (DOC). In order to determine the nitrogen mineralization rate (N_MIN_), soil samples were taken before and after 10 days of incubation at 30 °C. 

### 2.3. Statistical Analysis

Microsoft Excel 2019^®^ and Statistic 8.1^®^ were used to develop the aforementioned treatments using one-way ANOVA (Analysis of Variance) (Talhassee, USA’s Analytical Software). The dataset was compared using the least significant difference (LSD).

## 3. Results

### 3.1. Effect of Salicylic Acid (SA) and Farmyard Manure (FYM) on Root and Shoot Length of Maize Plant under Cd Stress

Data regarding root and shoot length of the maize plant were significantly affected by all studied treatments (Table 2). The highest root and shoot length (19.37 cm and 53.43 cm, respectively) was noticed in T_6_ when FYM and SA was applied without any Cd stress, followed by T_2_ (21.26 cm and 54.93 cm) when FYM was applied alone without Cd stress, and the lowest root and shoot length was noticed in T_5_ (11.33 cm and 44.13 cm) when Cd stress was at 200 mg/kg of soil.

### 3.2. Effect of SA and FYM on Plant Height of Maize under Cd Stress

Cadmium stress caused a significant reduction in maize plant height in all treatments carrying different concentrations of Cd stress. Application of farmyard manure (FYM) and salicylic acid (SA) significantly increased the plant height of maize crops. The highest plant height (77.68 cm) was observed in T_6_, indicating a 6% increase in growth when compared with the control, followed by T_2_ (76.19 cm), when FYM was applied alone without Cd stress, and the lowest plant height was noticed in T_5_ (55.46 cm), when Cd stress was at 200 mg/kg of soil. The application of farmyard manure mixed with salicylic acid, with Cd toxicity in the soil, significantly reduced the negative effect of Cd on the plant height of maize in all treatments (Table 2).

### 3.3. Effect of SA and FYM on Root, Shoot, and Plant Weight of Maize under Cd Stress

The root, shoot, and plant fresh weight were significantly affected by all studied treatments. The highest root, shoot, and plant fresh weight (10.95 g, 27.63 g, and 38.58 g) was noticed in T_6_, followed by T_2_ (10.27 g, 26.40 g, and 36.67 g), and the lowest results were noticed in T_5_ (3.42 g, 16.53 g, and 19.95 g). Greater decreases in root, shoot, and plant height were noticed when Cd toxicity increased. The highest plant dry weight (23.09 g) was noticed in T_6_ when FYM and SA were applied in combined form without any Cd stress, followed by T_2_ (22.38 g) when only FYM was applied without any Cd stress. The lowest plant dry weight (08.87 g) was observed in T_5_ when Cd stress was at 200 mg/kg of soil without FYM and SA (Table 3).

### 3.4. Effect of SA and FYM on Length and Number of Leaves of Maize under Cd Stress

Number of leaves and their length was significantly affected by the Cd toxicity and application of FYM and SA (Table 4). The greatest length of a leaf (17.82 cm) and number of leaves per maize plant (11.66) was noticed in T_6_ when there was no Cd toxicity and SA and FYM were applied in combined form, followed by T_2_ (16.54 cm and 10.66) when only FYM was applied, without any Cd toxicity, and the shortest length of the leaf and number of leaves was observed in T_5_ (08.92 cm and 1.66) when Cd toxicity was at 200 mg/kg of the soil without FYM and SA application. 

### 3.5. Effect of SA and FYM on Chlorophyll Content of Maize under Cd Stress

**Figure 1:**
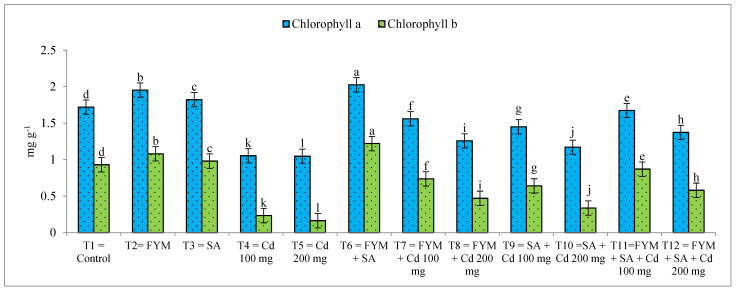
Effect of salicylic acid and farmyard manure on chlorophyll a&b of maize plant. Different letters on the bar shows significant differences between treatments.

The highest chlorophyll a&b (2.026 mg/g and 1.22 mg/g) was observed in T_6_ when SA and FYM were applied in combined form without Cd toxicity, followed by T_2_ (1.953 mg/g and 1.08 mg/g), when only FYM was applied, without any Cd toxicity, and the lowest chlorophyll a&b was observed in T_5_ (1.046 mg/g and 0.163 mg/g) when Cd toxicity was at 200 mg/kg of the soil, without FYM and SA application (Figure 1). 

### 3.6. Effect of SA and FYM on NPK Uptake of Maize under Cd Stress

**Figure 2:**
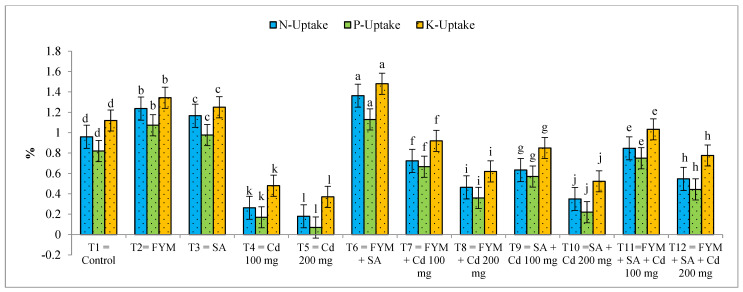
Effect of salicylic acid and farmyard manure on NPK uptake (%) of maize plant. Different letters on the bar show significant differences between treatments.

The application of farmyard manure and salicylic acid significantly increases the NPK uptake of maize and improves the plant growth conditions; thus, the average highest NPK uptake (%) was observed at T_6_. The NPK uptake of maize plants increased by 41%, 37%, and 32% (Figure 2). Cd stress caused a significant reduction in NPK uptake in maize plants in treatments that were carrying different concentrations of Cd alone, compared to T_6_ and other treatments that used Cd in combination with farm manure and salicylic acid. The lowest NPK uptake was observed at T_5_, carrying a concentration of 200 mg kg^−1^ of soil Cd, indicating a 90%, 84%, and 75% growth reduction, respectively.

### 3.7. Effect of SA and FYM on Crop Growth Rate (CGR) and Net Assimilation Rate (NAR) of Maize under Cd Stress

**Figure 3:**
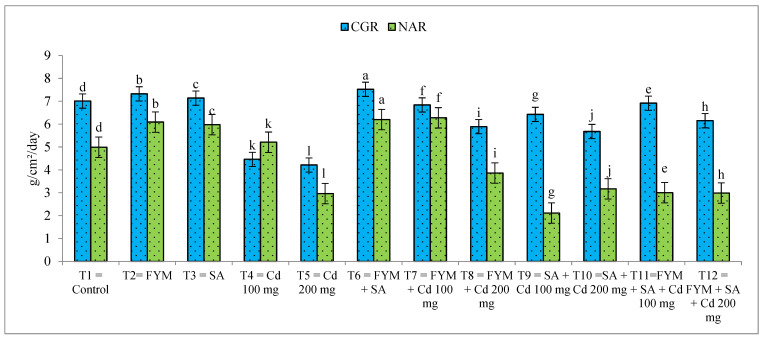
Effect of farmyard manure and salicylic acid on crop growth rate (CGR) and net assimilation rate (NAR) under cadmium stress condition. Different letters on the bar show significant differences between treatments.

Crop growth rate is a crucial indicator because it shows how well a crop is using input materials and creating the photosynthates that the plant uses to produce an economic yield. It was found that T_6_ (farmyard manure and salicylic acid) had the highest crop growth rate (7.533 g/cm^2^/day), followed by T_2_ (7.323 g/cm^2^/day). Crop growth rate decreased under Cd stress. The lowest CGR was found in T_5_, followed by T_4_, with Cd 200 mg/kg and Cd 100 mg/kg, respectively. The highest NAR was noticed in T_6_ (6.20 g/cm^2^/day), followed by T_2_ (6.09 g/cm^2^/day), and the lowest NAR was observed in T_5_ (2.96 g/cm^2^/day) (Figure 3).

### 3.8. Effect of SA and FYM on Soil Organic Carbon (SOC), Dissolved Organic Nitrogen (DON), Dissolved Organic Carbon (DOC), Nitrogen Mineralization (N_MIN_), and Soil Respiration (SR) of Maize-Grown Soil under Cd Stress

The highest SOC (1.540 g/kg), DOC (143.8 g/kg), DON (20.643 g/kg), N_MIN_ (72.853), and SR (25.627 g CO_2_/m^2^/day) was noticed in T_6_, when FYM and SA were applied in combined form, followed by F_2_ (SOC: 1.480 g/kg, DOC: 141.7 g/kg, DON: 19.463 g/kg, N_MIN_: 71.710, SR: 24.720 CO_2_/m^2^/day), when only FYM was applied, without any Cd stress, and the lowest results (SOC: 0.666 g/kg, DOC: 118.51 g/kg, DON: 9.620 g/kg, N_MIN_: 61.693, SR: 14.570 CO_2_/m^2^/day) were obtained in T_5_, when Cd stress was 200 mg/kg of soil (Table 5).

### 3.9. Effect of SA and FYM on Microbial Biomass Carbon (MBC), Phosphorus (MBP), and Nitrogen (MBN) of Maize-Grown Soil under Cd Stress

**Figure 4:**
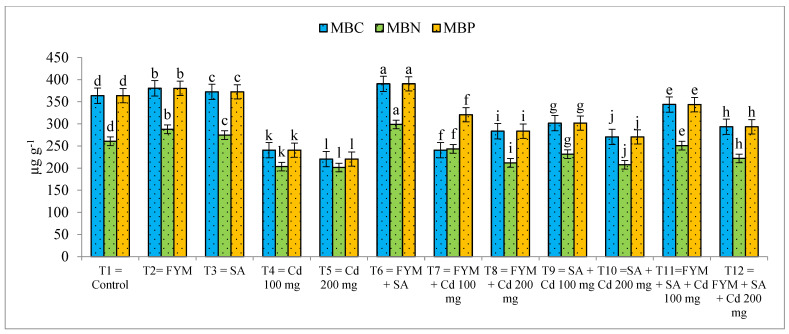
Effect of farmyard manure and salicylic acid on microbial biomass carbon (MBC), phosphorus (MBP), and nitrogen (MBN) under cadmium stress condition. Different letters on the bar show significant differences between treatments.

Data regarding the microbial biomass carbon (MBC), phosphorus (MBP), and nitrogen (MBN) show that Cd stress significantly reduces the results and that FYM application is more effective, as compared to SA, but the combined application of FYM with SA is more effective to control the negative effect of Cd toxicity (Figure 4). The highest MBC (390.49 µg g^−1^), MBN (298.64 µg g^−1^), and MBP (390.52 µg g^−1^) was noticed in T_6_ when FYM and SA were applied in combined form, followed by T_2_ (MBC: 380.48 µg g^−1^, MBN: 287.78 µg g^−1^, MBP: 380.27 µg g^−1^) when only FYM was applied, and the lowest results (MBC: 220.52 µg g^−1^, MBN: 201.28 µg g^−1^, MBP: 220.46 µg g^−1^) were observed in T_5_ when Cd stress was applied at 200 mg/kg of soil.

### 3.10. Effect of Farmyard Manure and Salicylic Acid on Soil pH, Mineral N, Bray P, and Exchangeable-K under Cadmium Stress Condition

Cd stress has negative effects on the soil chemical properties, but the combined application of FYM and SA is effective to control its negative impact. Soil pH, Mineral N, Bray P, and Exchangeable-K were significantly affected by all studied treatments (Table 6). The highest Soil pH (7.246), Mineral N (83.590 mg/kg), Bray P (7.846 mg/kg), and Exchangeable-K (173.58 mg/kg) was noticed in T_6_, followed by T_2_ (Soil pH: 7.136, Mineral N: 82.930 mg/kg, Bray P: 7.746 mg/kg, and Exchangeable-K: 172.52 mg/kg), and the lowest results were observed in T_5_ (Soil pH: 6.143, Mineral N: 68.397 mg/kg, Bray P: 6.446 mg/kg, and Exchangeable-K: 158.53 mg/kg).

## 4. Discussion

The use of farmyard manure combined with salicylic acid significantly improved and enhanced the vegetative growth of maize and mitigated the detrimental effect of Cd stress on maize growth. The findings highlight the beneficial properties of farmyard manure and salicylic acid in maize growth and development in environments where Cd contamination is a major problem, or even in the absence of Cd stress. Our findings are consistent with the previous studies of Sofy [33] and Bandyopadhyay [34] regarding the use of salicylic acids and farmyard manure in reducing the negative effects caused by heavy metals.

Salicylic acid can be used to diminish the negative effects of lead (Pb) toxicity on maize plants and to lessen oxidative damage [33]. According to Nasir et al. [35], applying salicylic acid, ascorbic acid, and farmyard manure significantly increased the amount of essential oils found in dragonhead by increasing the growth hormone levels in plants. In our investigation, the impacts of farmyard manure, alone, and in combination with salicylic acid, considerably improved many of the growth parameters of maize plants under a Cd-contaminated environment. The maize’s growth and yield characteristics were greatly enhanced compared to the control when FYM and SA were applied in combined form. Additionally, it was also noticed that the farmyard manure significantly enhanced the soil’s organic percentage. This increase in organic matter encourages the variety and growth of the microbial community in the soil’s rhizosphere [34,36]. Farmyard manure considerably boosted plant development indices while reducing the harmful impacts of Cd stress. Increase of root length density, root mass density, leaf area duration, and biomass help to increase nitrogen uptake, chlorophyll contents, and grain yield [34].

Salicylic acid application increased all growth features, including plant height, quantity, and area of green leaves, stem diameter, and dry weight of the stem, leaves, and entire plant. Tayyab et al. [37] found that foliar treatment of salicylic acid increased the growth parameters (plant height and dry weight) of maize seedlings under abiotic stress due to the increase of higher leaf area and growth hormone production. Zhang et al. [38] also found similar findings, such as that Cd stress lowers the root and plant height of maize in environments that suffer from soil contamination. However, using salicylic acid can enhance maize root and plant height growth under Cd stress [39]. Salicylic acid is shown to trigger the protective role from pathogens when its internal concentration is increased, which promotes a plant’s response, tolerance, and resistance to many ailments that damage plants [24]. Additionally, salicylic acid plays a variety of significant physiological tasks, including promoting protein synthesis, ion absorption, nutrition transfer, CO_2_ representation, ion absorption, blooming, and stomata movement [19]. Farmyard manure and salicylic acid applications were primarily responsible for this rise in plant height, shoot length, stem length, root length, and other physiological characteristics [34]. Providing macro- and micronutrients to plants, in the form of organic manures, helps to enhance the physical, chemical, and biological health of the soil [23]. Hayat et al. [40] concluded the same results for maize plant fresh and dry weight and reported that salicylic acid is found to enhance weight parameters more at a lower Cd stress level; at higher Cd stress levels, salicylic acid is found to be insufficient to mitigate the effect of Cd stress. FYM supplies vital nutrients, enhances soil structure, and lowers the amount of cadmium that is available in the soil [34]. When administered externally, salicylic acid stimulates the synthesis of antioxidants, controls hormones, and turns on defense mechanisms in maize [21,23,34]. These treatments work together to encourage root and shoot growth, which raises fresh and dry weights. Salicylic acid acts as a plant hormone that helps maize plants tolerate stress and activates defensive mechanisms [35,41]. Cd toxicity results in morphological parameter reductions and disturbances in the tissues of plants [42]. Cd stress decreases the cell division and photosynthesis in plants, which results in the reduction of root and shoot growth and plant height [43,44]. 

Photosynthetic characteristics are the major targets of cadmium’s harmful effects since they serve as major physiological indicators [45]. Similar outcomes for a reduction in the physiological characteristics of several plants caused by Cd stress have already been documented [46]. Cd toxicity in the soil causes harmful effects on plant cells and, ultimately, on chlorophyll, which results in Rubisco destruction and chlorophyll molecule disintegration [47]. Our results are also supported by the findings of many researchers who indicated that Cd reduced the root and shoot length which were enhanced by the application of salicylic acid [48,49,50]. Akhtar et al. [51] concluded similar results and indicated that high concentrations of Cd in agricultural soils resulted in the reduced growth of roots and shoots. 

The amount of soil organic carbon (SOC) is increased by SA and FYM. FYM, an organic material, increases the soil’s carbon content, whereas SA encourages the growth of root biomass, which increases carbon sequestration [52,53]. Higher SOC levels are the result of these actions, which are crucial for soil fertility and the health of the entire ecosystem [54]. SA and FYM have a favorable impact on the soil’s dissolved organic carbon (DOC) and dissolved organic nitrogen (DON). The release of root exudates, rich in organic compounds, is induced by SA treatment, increasing the availability of DON and DOC [55]. Since FYM is a source of nitrogen, it helps to increase the level of dissolved organic nitrogen [23,33]. In the presence of cadmium stress, these variations in DON and DOC concentrations increase nutrient availability and stimulate microbial activity, resulting in improved soil fertility and nutrient cycling [33]. The release of mineral nitrogen from organic matter is boosted as a result of SA’s promotion of the synthesis of enzymes involved in nitrogen mineralization [55,56]. FYM serves as a substrate for microbial activity and furthers N_MIN_ since it is rich in organic nitrogen [57]. Additionally, SA and FYM both contribute to increased soil respiration, indicating increased microbial activity and nutrient turnover, both of which are very effective for sustaining plant growth and soil health in the presence of cadmium stress [52,55,57].

SA and FYM can increase the soil’s microbial biomass [52,55,57]. SA stimulates root exudation by supplying a carbon-rich substrate that fosters microbial activity and growth [52]. FYM provides an ideal habitat for microorganisms to flourish because it is organic matter [33,55]. A higher number of microorganisms, which are essential for the cycling of nutrients and the health of the soil’s ecosystem, is shown by the increased microbial biomass carbon [58,59]. In order to increase the availability of phosphorus to soil microorganisms, SA treatment can improve the release of organic phosphorus compounds through root exudation and enzymatic activities [60]. For microbial growth, FYM contributes to the supply of organic nitrogen by creating a nitrogen-rich environment [28]. As a result, the microbial biomass’s phosphorus and nitrogen levels rise, showing that, under the influence of cadmium stress, nutrient availability and cycling have improved [33].

Farmyard manure is a rich source of nutrients and organic materials that can change the pH of soil [33]. When soil is exposed to Cd stress, organic matter from FYM decomposes, releasing organic acids that can help buffer the pH and keep the soil from becoming overly acidic [33,39,41]. By enhancing cation exchange capacity and improving soil structure, organic matter in FYM can also affect soil pH by minimizing the leaching of acidic chemicals [61]. Salicylic acid also indirectly affects soil pH by encouraging root development and the exudation of organic substances, which may also increase the soil’s ability to act as a buffer [62]. The mineralization of FYM’s organic nitrogen by soil bacteria results in the release of other forms of nitrogen, such as ammonium (NH4+) and nitrate (NO3-) [63]. This may make more nitrogen available for plant uptake when exposed to cadmium stress. Similar to this, salicylic acid may improve maize plants’ ability to absorb nitrogen by promoting root growth and nutrient uptake [62]. This would lead to a higher level of mineral nitrogen in the soil. Phosphorus is present in farmyard manure, and it can be gradually released through mineralization, leading to greater Bray P levels. Additionally, FYM’s organic matter helps plants gain and retain potassium from the soil [63]. Salicylic acid has the ability to increase nutrient uptake and raise nutrient usage efficiency, which may result in more phosphorus and potassium building up in the soil [62].

## 5. Conclusions

Cadmium (Cd) toxicity in the soil caused a significant decrease in maize crop growth and damaged the soil’s rhizospheric properties. The higher dose of Cd negatively affected maize crop growth and productivity. However, using salicylic acid (SA) and farmyard manure (FYM) resulted in effective results, in terms of improving maize crop growth and soil properties. Both SA and FYM mitigated Cd stress in maize crop with the surpass of combined application in most of the traits. Future research needs regarding the mechanisms involved in the application of salicylic acid on plants grown under different stress conditions include its fate in the environment and consequences for soil, plant, animal, and human health.

## Figures and Tables

**Table 1:** Characteristics of the experimental soil.

Physiochemical Properties of Soil	Values
Ph	6.88
EC	4.97 dSm^−1^
Organic matter	4.93%
Moisture content	19.01%
Concentration of nutrients	
Potassium K	44.49 mg L^−1^
Sodium Na	25.01 mg L^−1^
Magnesium Mg	22.30 mg L^−1^
Calcium Ca	168.69 mg L^−1^
Soil composition percentage	
Clay	50%
Sand	25%
Silt	25%

**Table 2:** Effect of farmyard manure and salicylic acid on root and shoot length and plant height of maize under cadmium stress.

Treatments	Root Length (cm)	Shoot Length (cm)	Plant Height (cm)
T_1_ = Control	19.37 d	52.43 d	71.81 d
T_2_ = Farmyard manure	21.26 b	54.93 b	76.19 b
T_3_ = Salicylic acid	20.73 c	53.15 c	73.88 c
T_4_ = Cadmium (100 mg/kg)	12.43 k	45.55 k	57.98 k
T_5_ = Cadmium (200 mg/kg)	11.33 l	44.13 l	55.46 l
T_6_ = Farmyard manure + Salicylic acid	22.43 a	55.25 a	77.68 a
T_7_ = FYM + Cd (100 mg/kg)	17.92 f	50.74 f	50.74 f
T_8_ = FYM + Cd (200 mg/kg)	14.57 i	47.15 i	47.15 i
T_9_ = Salicylic acid + Cd (100 mg/kg)	16.63 g	49.22 g	49.22 g
T_10_ = Salicylic acid + Cd (200 mg/kg)	13.64 j	46.64 j	60.28 j
T_11_ = FYM + Salicylic acid + Cd (100 mg/kg)	18.35 e	51.28 e	69.63 e
T_12_ = FYM + Salicylic acid + Cd (200 mg/kg)	15.72 h	48.23 h	48.23 h

Means that do not have a common letter show significant differences at a 5% probability level.

**Table 3:** Effect of farmyard manure and salicylic acid on plant weight (g) of maize under cadmium stress.

Treatments	Root Fresh Weight (g)	Shoot Fresh Weight (g)	Plant Fresh Weight (g)	Plant Dry Weight (g)
T_1_ = Control	08.75 d	24.43 d	33.18 d	19.26 d
T_2_ = Farmyard manure	10.27 b	26.40 b	36.67 b	22.38 b
T_3_ = Salicylic acid	09.87 c	25.26 c	35.14 c	21.08 c
T_4_ = Cadmium (100 mg/kg)	03.95 k	17.66 k	21.61 k	10.39 k
T_5_ = Cadmium (200 mg/kg)	03.42 l	16.53 l	19.95 l	08.87 l
T_6_ = Farmyard manure + Salicylic acid	10.95 a	27.63 a	38.58 a	23.09 a
T_7_ = FYM + Cd (100 mg/kg)	06.73 f	22.43 f	29.16 f	17.21 f
T_8_ = FYM + Cd (200 mg/kg)	05.65 i	19.56 i	25.21 i	13.12 i
T_9_ = Salicylic acid + Cd (100 mg/kg)	06.45 g	21.66 g	28.12 g	15.93 g
T_10_ = Salicylic acid + Cd (200 mg/kg)	04.66 j	18.26 j	22.93 j	12.10 j
T_11_ = FYM + Salicylic acid + Cd (100 mg/kg)	07.84 e	23.73 e	31.58 e	18.38 e
T_12_ = FYM + Salicylic acid + Cd (200 mg/kg)	06.14 h	20.66 h	26.80 h	14.31 h

Means that do not have a common letter show significant differences at a 5% probability level.

**Table 4:** Effect of salicylic acid and farmyard manure on length of leaf (cm) and number of leaves (n) of maize.

Treatments	Length of Leaf (cm)	Number of Leaves (n)
T_1_ = Control	14.65 d	08.66 d
T_2_ = Farmyard manure	16.54 b	10.66 b
T_3_ = Salicylic acid	15.94 c	10.66 c
T_4_ = Cadmium (100 mg/kg)	09.65 k	01.66 k
T_5_ = Cadmium (200 mg/kg)	08.92 l	01.66 l
T_6_ = Farmyard manure + Salicylic acid	17.82 a	11.66 a
T_7_ = FYM + Cd (100 mg/kg)	13.14 f	06.66 f
T_8_ = FYM + Cd (200 mg/kg)	10.46 i	03.66 i
T_9_ = Salicylic acid + Cd (100 mg/kg)	12.86 g	05.66 g
T_10_ = Salicylic acid + Cd (200 mg/kg)	10.12 j	02.66 j
T_11_ = FYM + Salicylic acid + Cd (100 mg/kg)	13.43 e	07.66 e
T_12_ = FYM + Salicylic acid + Cd (200 mg/kg)	13.14 h	04.66 h

Means that do not have a common letter show significant differences at a 5% probability level.

**Table 5:** Farmyard manure and salicylic acid affect SOC, DOC, DON, N_MIN_, and SR under cadmium stress.

Treatments	SOC (g kg^−1^)	DOC (g kg^−1^)	DON (g kg^−1^)	N_MIN_	SR (g CO_2_/m^2^/day)
T_1_ = Control	1.333 d	137.6 d	17.663 d	69.740 d	22.77 d
T_2_ = Farmyard manure	1.480 b	141.7 b	19.463 b	71.710 b	24.72 b
T_3_ = Salicylic acid	1.380 c	140.7 c	18.857 c	70.77 c	23.66 c
T_4_ = Cadmium (100 mg/kg)	0.740 k	120.3 k	10.823 k	62.287 k	15.69 k
T_5_ = Cadmium (200 mg/kg)	0.666 l	118.5 l	09.620 l	61.693 l	14.57 l
T_6_ = Farmyard manure + Salicylic acid	1.540 a	143.8 a	20.643 a	72.853 a	25.62 a
T_7_ = FYM + Cd (100 mg/kg)	1.220 f	135.5 f	15.857 f	67.493 f	20.37 f
T_8_ = FYM + Cd (200 mg/kg)	0.933 i	128.7 i	12.500 i	64.737 i	17.62 i
T_9_ = Salicylic acid + Cd (100 mg/kg)	1.180 g	133.6 g	14.773 g	66.557 g	19.52 g
T_10_ = Salicylic acid + Cd (200 mg/kg)	0.856 j	126.7 j	11.407 j	63.243 j	16.53 j
T_11_ = FYM + Salicylic acid + Cd (100 mg/kg)	1.280 e	136.6 e	16.467 e	68.460 e	21.61 e
T_12_ = FYM + Salicylic acid + Cd (200 mg/kg)	1.080 h	130.6 h	13.827 h	65.323 h	18.52 h

Means that do not have a common letter show significant differences at a 5% probability level.

**Table 6:** Effect of farmyard manure and salicylic acid on Soil pH, Mineral N, Bray P, and Exchangeable-K under cadmium stress condition.

Treatments	Soil pH	Mineral N (mg kg^−1^)	Bray P (mg kg^−1^)	Exchangeable-K (mg kg^−1^)
T_1_ = Control	6.960 d	80.04 d	7.526 d	170.77 d
T_2_ = Farmyard manure	7.136 b	82.93 b	7.746 b	172.52 b
T_3_ = Salicylic acid	7.026 c	81.64 c	7.640 c	171.59 c
T_4_ = Cadmium (100 mg/kg)	6.213 k	69.49 k	6.536 k	159.23 k
T_5_ = Cadmium (200 mg/kg)	6.143 l	68.39 l	6.446 l	158.53 l
T_6_ = Farmyard manure + Salicylic acid	7.246 a	83.59 a	7.846 a	173.58 a
T_7_ = FYM + Cd (100 mg/kg)	6.743 f	78.79 f	7.343 f	168.53 f
T_8_ = FYM + Cd (200 mg/kg)	6.436 i	74.48 i	6.960 i	165.48 i
T_9_ = Salicylic acid + Cd (100 mg/kg)	6.653 g	77.60 g	7.256 g	167.84 g
T_10_ = Salicylic acid + Cd (200 mg/kg)	6.340 j	73.69 j	6.853 j	163.68 j
T_11_ = FYM + Salicylic acid + Cd (100 mg/kg)	6.836 e	79.55 e	7.466 e	169.75 e
T_12_ = FYM + Salicylic acid + Cd (200 mg/kg)	6.563 h	76.45 h	7.153 h	166.51 h

Means that do not have a common letter show significant differences at a 5% probability level.

## Data Availability

Not applicable.
